# Vibration and Bandgap Behavior of Sandwich Pyramid Lattice Core Plate with Resonant Rings

**DOI:** 10.3390/ma16072730

**Published:** 2023-03-29

**Authors:** Chengfei Li, Zhaobo Chen, Yinghou Jiao

**Affiliations:** School of Mechatronics Engineering, Harbin Institute of Technology, Harbin 150001, China

**Keywords:** sandwich plate, elastic metamaterials, bandgap, vibration properties

## Abstract

The vibration suppression performance of the pyramid lattice core sandwich plates is receiving increasing attention and needs further investigation for technical upgrading of potential engineering applications. Inspired by the localized resonant mechanism of the acoustic metamaterials and considering the integrity of the lattice sandwich plate, we reshaped a sandwich pyramid lattice core with resonant rings (SPLCRR). Finite element (FE) models are built up for the calculations of the dispersion curves and vibration transmission. The validity of the bandgap of the SPLCRR and remarkable vibration suppression are verified by experimental observations and the numerical methods. Furthermore, the effects of geometric parameters, material parameters and period parameters on the bandgaps of the SPLCRR are systematically investigated, which offers a deeper understanding of the underlying mechanism of bandgap and helps the SPLCRR structure meet the technological update requirements of practical engineering design.

## 1. Introduction

The continuous development and technological update requirements of aerospace platforms and high-speed rails have led to great efforts for designing new structures and materials that integrate both lightweight and special multifunctional properties [1,2,3,4,5,6]. However, sandwich plates generate harmful vibration and radiated noise that can negatively impact both equipment functionality and operator safety. Therefore, it is crucial to explore innovative methods and theories for controlling vibration and noise in plate structures. The lattice sandwich structure is a multi-layer structure consisting of two panels and a truss core. In recent years, the lattice sandwich structures attract huge attention due to their high stiffness, low density and light weight [7,8,9,10,11]. Interestingly, various grid-truss core structures such as tetrahedron, three-dimensional kagome, and vertebral body structures have been developed [7,11]. At present, there are many studies on mechanical properties, manufacturing process, vibration characteristics, and dynamic responses of lattice sandwich structures [12,13,14]. Nevertheless, the elastic wave propagates at lower frequencies due to the open cell sandwich structure and high stiffness to mass ratio, leading to poor vibration attenuation and acoustic performance [8,10]. The low-frequency mode contributes the most to sound power, and thus, effective reduction in sound power can be achieved by suppressing vibrations in the low-frequency range [15]. Therefore, it is still a challenge for lattice sandwich structures to simultaneously achieve the balance of vibration suppression and light weight.

Fortunately, in 2000, Liu and co-workers [16] proposed a localized resonant mechanism, also known as elastic metamaterials (EM), to break the conventional mass-density law of sound transmission, significantly improving sound transmission loss (STL) below 400 Hz. Since then, numerous studies have been carried out relating to the properties of unusual structures, such as negative modulus [17,18,19], energy harvesting [20,21,22], negative refraction [23], acoustic invisibility [24,25], vibration attenuation [26,27,28,29,30], topological acoustics [31] and so forth, which could enhance wave modulation of classical lattice sandwich structures.

Nowadays, there is plenty of research on the propagation of flexural waves and band structures in thin plates. It is noteworthy that the structures showed mechanisms of low frequency forbidden bands and Bragg bandgaps in parallel with local resonance and Bragg scattering. The bandgaps, in turn, are directly influenced by the geometry and lattice symmetry of the resonator array [32]. Yong et al. [33] extended the well-known plane wave expansion method (PWEM) to deal with plate systems with periodic arrays of spring-mass resonators, and they determined that the bandwidth of the bandgaps is greatly affected by the frequencies of the causes of the local resonators. Furthermore, considering the Kirchhoff–Love thin-plate theory, Miranda et al. investigated the band structure of flexural waves propagating in thin metamaterial plates and the effect of periodic arrays of multi-degree-of-freedom local resonators in square and triangular lattices [34].

Using finite element model calculations, Massimo et al. [35] investigated the dynamic response of a three-layer sandwich panel with a honeycomb core. They also investigated the effect of individually periodic placement of cores of different geometries in the two-dimensional space of the structure. Song et al. [36] studied the sound transmission of sandwich panels and its reduction using the bandgap concept. The results showed that sound transmission was significantly reduced over the bandgaps of periodic sandwich panels. Li and An [27,37] proposed improved three-dimensional truss lattice structures for low and broadband elastic wave absorption, respectively.

The abovementioned works are concerned with the bandgap, vibration reduction, and acoustic characteristics of various plated structures. Although some achievements have been chosen in resolving the vibration attenuation and acoustic characteristics of thin or sandwich plates by attaching mass block, the uniformity of lattice sandwich plates was challenged [27]. In this work, inspired by the localized resonant mechanism of the acoustic metamaterials and considering the integrity of the lattice sandwich plate, we reshaped a sandwich pyramid lattice core with resonant rings (SPLCRR). Compared to the typical lattice sandwich plate, the SPLCRR addition of a resonant ring onto the rod enables the sandwich plate to fulfill the requirements for a smooth outer surface in engineering applications while also providing increased stiffness, structural simplicity, and ease to design. The dispersion curves of the SPLCRR and evolution of the bandgaps for the SPLCRR are investigated to achieve a wide bandgap in the low-frequency range. The effect of bandgaps on vibration attenuation is further investigated by theoretical and experimental verification. Furthermore, the effects of geometric parameters, material parameters and period parameters on the bandgap of the SPLCRR are systematically analyzed, which offers a deeper understanding of the underlying mechanism of bandgap and helps the SPLCRR structure meet the technological update requirements of practical engineering design.

## 2. Model and Theory

**Figure 1:**
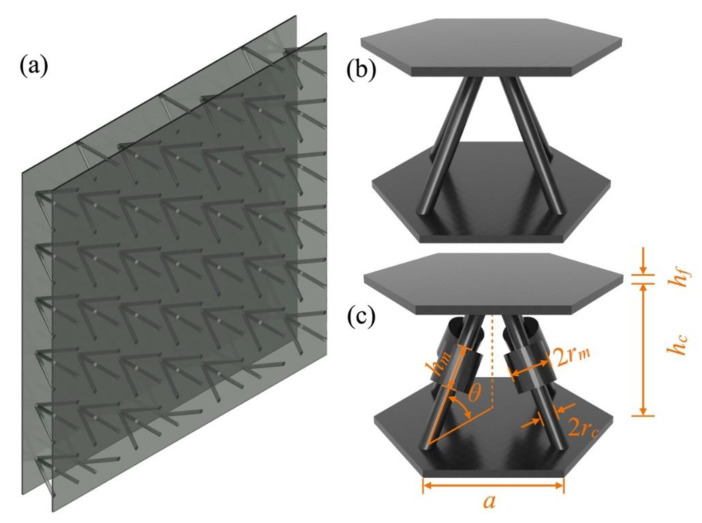
(**a**) Typical lattice sandwich plate, (**b**) typical lattice sandwich unit cell, (**c**) SPLCRR unit-cell.

Figure 1a illustrates a diagram depicting a typical lattice sandwich structure plate with periodic lattice-truss-core. As shown in Figure 1b,c, the typical lattice unit cell and the SPLCRR unit cell are considered in this work, respectively. Different from the system of the traditional elastic metamaterial (EM) unit cell which is usually made by attaching different materials to the surface of the plate, the unit cell of SPLCRR has two radius jump discontinuities in the core rods. The equation that governs the propagation of elastic waves in solids is expressed as follows:

(1) $$\sum_{j=1}^{3}\left\{\frac{\partial }{\partial x}\left(\lambda \frac{\partial u_{j}}{\partial x_{j}}\right)+\frac{\partial }{\partial x_{j}}\left[\mu \left(\frac{\partial u_{i}}{\partial x_{j}}+\frac{\partial u_{j}}{\partial x_{i}}\right)\right]\right\}=\rho \frac{\partial^{2}u_{i}}{\partial t^{2}};i,j=x,y,z,$$

where *r* represents the mass density, *u* and *v* denote the displacement vector, *t* denotes time, and *λ* and *μ* represent the Lame constants. Additionally, *x*, *y* and *z* represent the Cartesian coordinate variables.

In order to investigate the bandgaps of the proposed SPLCRR plate, a series of calculations of dispersion relations are conducted with the FEM based on Solid Mechanics Module of the COMSOL. Based on the periodicity of the proposed plate, we assumed that in both the *x* and *y* directions, single unit cell was considered, as depicted in Figure 1c. An adaptive mesh was selected to achieve sufficiently good convergence while minimizing computational costs within a reasonable time constraint.

Due to the Bloch–Floquet theorem, interface between adjacent cells using Bloch periodic boundary conditions can be expressed as follows:

(2) $$u\left(r\right)=u_{k}\left(r\right)e^{ikr},$$

where *u* is the displacement and *r* refers to the coordinate vectors.

**Figure 2:**
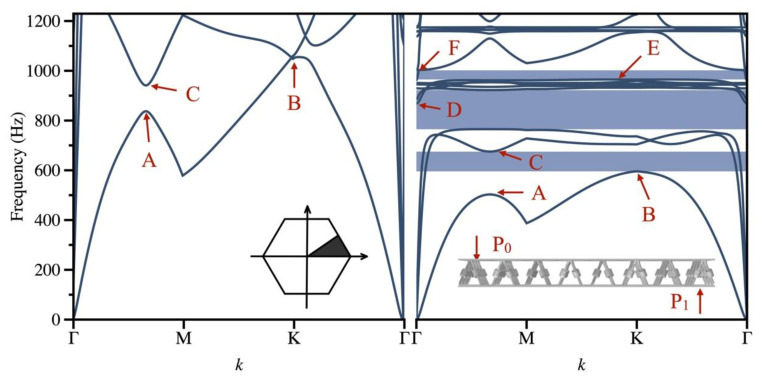
Dispersion curves of the sandwich pyramid lattice core plates without and with resonant rings on the left and right panels, respectively. Inset: The left panel is the first irreducible Brillouin zone, and the right panel is the input/output setting of numerical simulation.

After scanning all Bloch wave vectors along the edge of ΓXM in various lattice unit cells, the dispersion curves for the periodic sandwich plates can be obtained separately. By modulating the wave vector k within the first irreducible Brillouin zone (as depicted in the left inset of Figure 2) of the unit cell [30], one can derive the dispersion curves of the unit cell with respect to its wave number and frequency. These curves facilitate the determination of the band structure of an infinite periodic plate, which in turn enables the identification of its bandgaps.

To further verify the bandgap properties of the SPLCRR, the vibration attenuation characteristics of this plate were calculated by FEM. As shown in the inset on the right panel of Figure 2, the sandwich plate comprises 7 × 7 cells, a harmonic excitation is loaded at the edge of the panel, and the response occurs on the other side of the panel. It is assumed that the deflection of the sandwich panel under the transverse load is far less than its thickness. As a result, this study only takes into account the out-of-plane bandgaps and disregards the displacement parameters in the x-y plane.

At point P_0_ of the plate, an input acceleration excitation (denoted as *a_in_* ) is introduced, and the resulting acceleration response (denoted as *a_out_*) is measured at the response acquisition point P_1_, as depicted in the inset on the right of Figure 2. Ultimately, the vibration attenuation *T* can be obtained by altering the frequency of the input acceleration excitation:

(3) $$T=20log\frac{\left|a_{out}\right|}{\left|a_{in}\right|}.$$

## 3. Results and Discussion

In this section, a series of numerical simulations are conducted to analyze the dispersion relations properties of the proposed SPLCRR. The dispersion relations and vibration attenuation of the SPLCRR design matched up with the typical lattice sandwich plate, respectively. The influence of geometric parameters, material parameters, and period parameters on bandgap characteristics is systematically discussed.

### 3.1. Simulation Parameters

As shown in Figure 1c, the SPLCRR is composed of two face-sheets and a core layer which is four diagonals with additional resonant rings, wherein the sandwich plate with truss core is characterized by the total thickness represented by *h*, where the upper and lower face sheets possess equal thickness *h_f_*‘, and the core height is denoted by *h_c_*. The length, radius and inclination angle of the truss core and the resonant ring are represented by *l*, *h_m_*, *r_c_*, *r_m_* and *θ*, respectively. The geometrical parameters and material properties of the model are listed in Table 1 and Table 2.

Figure 2 presents the calculated dispersion curves of the proposed SPLCRR and the typical lattice sandwich plate. According to plate wave theory, a finite thickness plate can support the propagation of various of anti-symmetric (A-mode) and symmetric (S-mode) Lamb waves and shear-horizontal (SH-mode) waves with cutoff frequencies, including three fundamental plate modes. Compared with the typical lattice sandwich plate, the band structure of the SPLCRR presents a complete bandgap from 780 Hz to 990 Hz (the dark blue region in Figure 2) and a flexural wave bandgap from 600 Hz to 660 Hz (the light blue region in Figure 2). In a complete bandgap, the propagation of elastic waves will be significantly suppressed. Regarding various localized modes of guided elastic waves in solids, the dispersion curves of longitudinal and shear modes remain straight since their wave velocity is constant across frequencies. Conversely, the dispersion curves of flexural waves are non-linear because the wave velocity is dependent on frequency. Generally, flexural waves carry more vibrational energy than other types of waves. As a result, the complete flexural-wave bandgap can effectively reduce vibrations [34].

**Figure 3:**
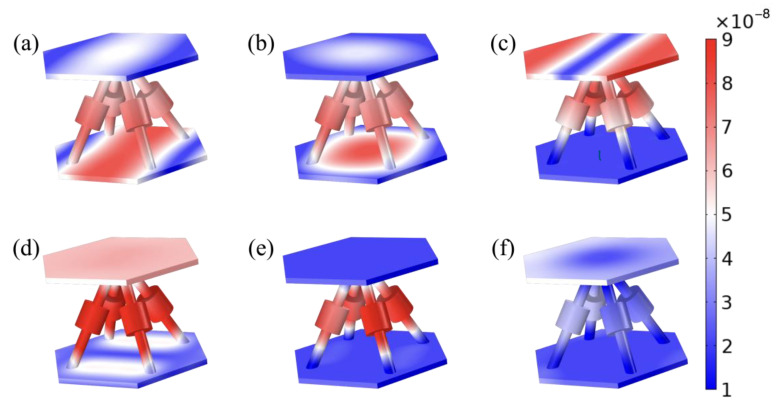
Eigenmodes of the SPLCRR unit cell at frequency (**a**) *f*_A_ = 502 Hz (**b**) *f*_B_ = 596 Hz (**c**) *f*_C_ = 625 Hz (**d**) *f*_D_ = 874 Hz (**e**) *f*_F_ = 964 Hz and (**f**) *f*_E_ = 1003 Hz.

To further illustrate the physical mechanism of the bandgap, the eigenmodes of the two models at the bandgap boundary frequencies were analyzed and are shown in Figure 3. The frequencies A, B and C are located on the first and second order dispersion curves. As depicted in Figure 2, the SPLCRR reduces the frequencies of the eigenmodes and opens and widens the bandgap at point B. More complex coupling effects are obtained for the eigenmodes at the upper and lower edges of the bending wave bandgap.

As depicted in Figure 2, the eigenmodes at frequencies D, F represent the upper edge of the complete bandgap and the flexural wave bandgap, which are at fourth and ninth dispersion curves, respectively. The intermediate state E_1_ and E_2_ represent the eigenmodes of the fourth to eighth dispersion curve. The face-sheets of the SPLCRR primarily exhibit lateral motion, with negligible movement in the vertical direction. The motion characteristics of the complete bandgap and the flexural wave bandgap will significantly affect vibration attenuation, as we will show later.

**Figure 4:**
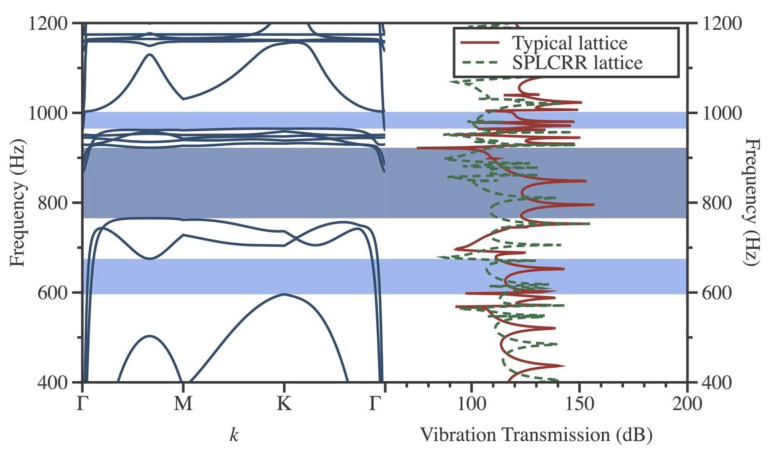
Vibration transmission spectra of non-dissipative typical and SPLCRR structures.

To obtain vibration attenuation, one can calculate T by sweeping a range of excitation frequencies. Figure 4 shows a comparison of the vibration attenuation calculated for a typical lattice sandwich plate and SPLCRR. The dispersion spectra yield two distinct bandgaps, represented by dark blue and light blue regions, corresponding to the complete bandgap and the flexural-wave bandgap, respectively. The vibration attenuation in the SPLCRR is significantly higher than in the typical lattice sandwich plate, both within the complete bandgap and the flexural-wave bandgap (represented by the light blue regions). Moreover, the vibration attenuation bands agree with the bandgaps shown in the dispersion curve. In contrast, the flexural-wave bandgap in the other region (680 Hz–760 Hz) does not produce significant vibration attenuation. Corresponding to the eigenmodes (see Figure 3) of the upper and lower boundaries of this bandgap, it can be seen that the suppression of out-of-plane vibrations does not play a significant role.

### 3.2. Experimental Verification

In Section 3.1, we calculated the vibration transmission of a typical lattice plate and a SPLCRR plate using FEM and compared it with their dispersion curve to verify the bandgaps and the performance of the vibration attenuation. To further verify the validity of the bandgaps and vibration attenuation performance, in this section, a typical lattice plate and a SPLCRR plate are used for experimental verification.

**Figure 5:**
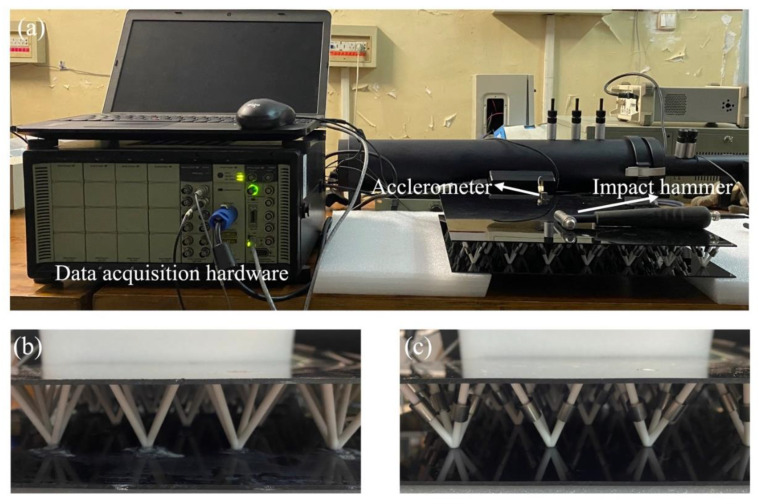
(**a**) The experiment set-up (**b**) typical sandwich plate (**c**) SPLCRR plate.

**Figure 6:**
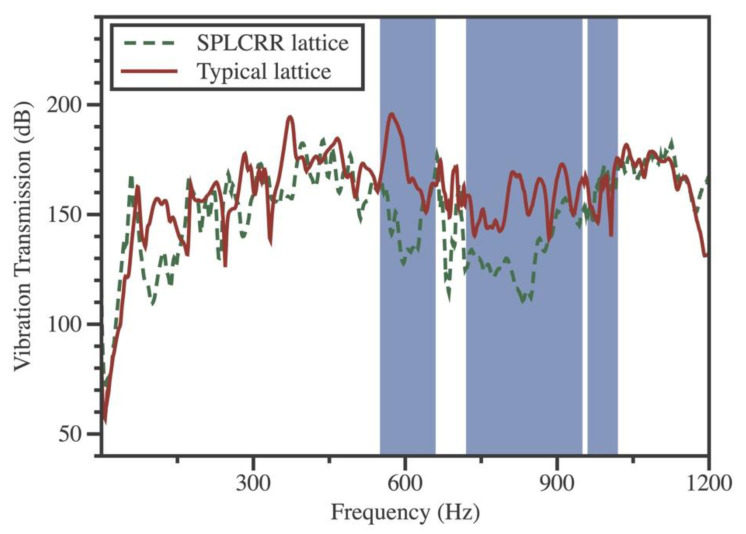
Comparison between the typical sandwich plate and SPLCRR plate for vibration transmission spectra.

The specimens contain 7 × 7 units (360 mm × 360 mm) with the same unit geometry and material parameters as in the FEM in Section 3.1. The experimental setup and experimental specimens are shown in Figure 5. Two end sides of the specimen are simply supported by foam materials to mimic the simply supported boundary condition. The vibration transmission spectra of the sandwich plates specimen were obtained by collecting the impact hammer (Brüel and Kjær, 8206-002) of the exciting point and the vibration acceleration (Brüel and Kjær, 4507) responses of the acquisition point with date acquisition (Brüel and Kjær, 3040). The numerical bandgaps detected in Figure 6 are represented by the blue regions. It was observed that the measured vibration suppression ranges exhibited a strong agreement with both the predicted bandgaps and the previous FEM simulation. The vibration suppression performance is experimentally verified in the proposed SPLCRR plate.

### 3.3. Effect of Geometric Parameters on Bandgap

Structural geometric parameters are one of the main factors affecting the bandgap of metamaterials. To further study the effect of geometric parameters on the bandgap, the dispersion curves of the SPLCRR are calculated under different rod diameters, angles between rods and panels, panel thicknesses, oscillator diameters, and oscillator lengths. Throughout the study, unless explicitly mentioned, the material and geometry parameters employed are the same as those specified in Section 3.1.

**Figure 7:**
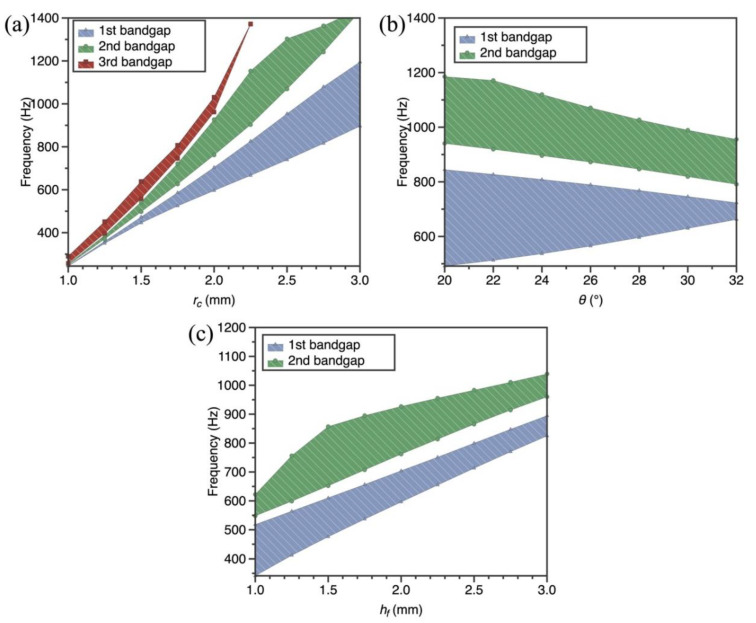
Effect of geometric parameters of lattice core and face-sheets on bandgaps of the SPLCRR. (**a**) lattice core rod radius values *r_c_* (**b**) angle values *θ* (**c**) face-sheet thickness values *h_f_*.

In this section, we present an analysis of bandgap behaviors of SPLCRR with varying lattice core and face-sheets dimensions. The investigation encompasses nine discrete lattice core rod radius values (*r_c_* = 1 mm–3 mm) and angle values (*θ* = 20–32°), and face-sheet thickness values (*h_f_* = 2 mm–3 mm) are chosen. The bandgap behaviors of SPLCRR are significantly influenced by changes in the lattice core and face-sheet dimensions, as evident from Figure 7, wherein the bandgaps move to higher frequencies with the increase in the rod radius and face-sheets thickness, but the increase in the lattice core rod angle causes the bandgaps to move to low frequencies. This is expected, because the change in lattice core parameters and the face-sheets parameter results in a modification stiffness of lattice sandwich layer (eq. X). It is worth noting that the width of the second bandgap of our interest reaches its maximum when *r_c_* = 2.5 mm, *θ* = 22° and *h_f_* = 2.5 mm, respectively. In addition, the change in rod radius is more sensitive to width of the second bandgap as compared to other two parameters.

**Figure 8:**
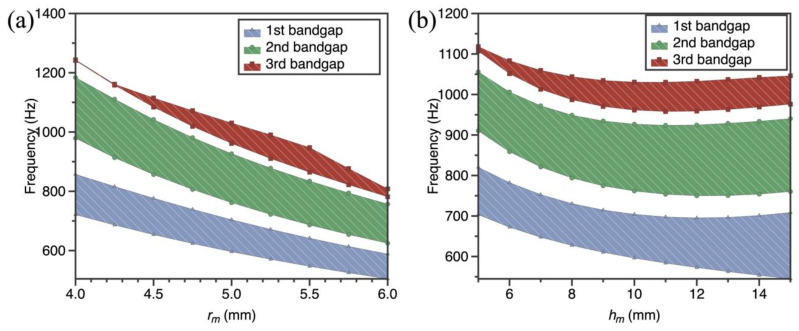
Effect of geometric parameters of resonant ring on bandgap of the SPLCRR. (**a**) resonant ring radius values *r_m_* (**b**) resonant ring height values *h_m_*.

As the dimensions of resonant ring are regarded as highly significant parameters in the whole SPLCRR, it is essential to evaluate its effect on the bandgap behaviors of SPLCRR. Thus, the influences of two geometric parameters *r_m_* and *h_m_* (defined in Section 3.1) of resonant ring on bandgaps are discussed in detail here, where nine different geometric parameters (*r_m_* = 4–6 mm) and (*h_m_* = 5–15 mm) chosen in this example. From Figure 8, it can be observed that the bandgap behaviors are strongly influenced by resonant ring radius and height, and increasing the radius and height leads to the bandgaps moving to lower frequency. This is expected, because the increase in resonant ring radius and height results in a modification of equivalent mass of lattice sandwich layer. In addition, the change in the resonant ring radius and height has no significant effect on the width of the second bandgap. In other words, the bandgap frequency range can be adjusted by changing geometric parameters of the resonant ring without affecting the bandgaps width.

### 3.4. Effect of Material Parameters on Bandgap

Material parameters are one of the main factors affecting the bandgap of metamaterials. To further investigate the effect of material parameters on the bandgap, the dispersion curves of the SPLCRR were calculated for different rod, panel, resonant ring elastic modulus and resonant ring damping ratio.

**Figure 9:**
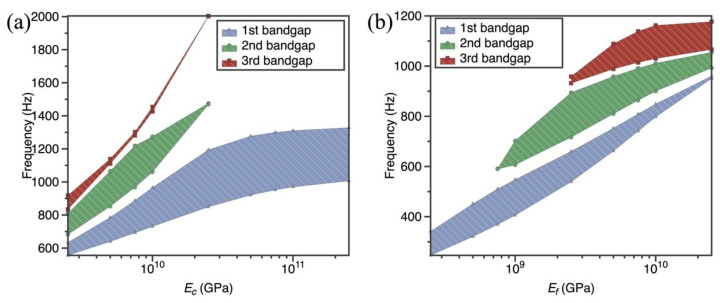
Effect of material parameters of lattice core and face-sheets on bandgaps of the SPLCRR. (**a**) elastic modulus of lattice rod *E_c_* (**b**) elastic modulus of face-sheets *E_f_*.

As can be seen in Figure 9, nine different elastic modulus of lattice rod (*E_c_* = 2.5–250 GPa) and face-sheets (*E_f_* = 0.25–25 GPa) are chosen in this example; the other properties are the same as those in Section 3.1. From Figure 9, it can be observed that the change in the elastic modulus of lattice rod and face-sheets exerts a significant impact on the bandgap behaviors of SPLCRR, wherein the bandgaps move to higher frequencies with the increase in the elastic modulus of lattice rod and face-sheets. This is reasonable that the increase in the elastic modulus of lattice rod and face-sheets results in the increase in equivalent stiffness of lattice sandwich layer. It is worth noting that the width of the second bandgap of our interest reaches its maximum when *E_c_* = 7.5 GPa and *E_f_* = 2.5 GPa, respectively. In addition, the width of the second bandgap first increases and then gradually disappears with the increase in the elastic modulus *E_c_*, while the increase in the elastic modulus *E_f_* shows the opposite trend.

**Figure 10:**
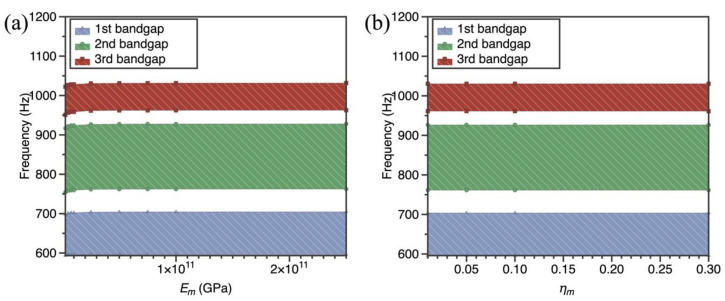
Effect of material parameters of resonant ring on bandgaps of the SPLCRR. (**a**) elastic modulus of resonant ring *E_m_* (**b**) damping ratio of the resonant ring *η_m_*.

As the material parameters of resonant rings are regarded as highly significant parameters in the whole SPLCRR, it is essential to evaluate its effect on the bandgap behaviors of SPLCRR. Thus, the influence of two material parameters *E_m_* and *η_m_* (defined in Section 3.1) of resonant ring on bandgaps are discussed in detail here, where nine different material parameters (*E_m_* = 2.5–250 GPa) and (*η_m_* = 0.01–0.3) are chosen in this example. From Figure 10, it can be obtained that with the increase of Young's modulus and damping ratio of the resonant ring, there is no significant change in the upper and lower edge frequencies of the band gap and no significant change in the bandgap width. Therefore, the Young's modulus and damping ratio of the resonant ring has little effect on the band gap of the metamaterial plate.

### 3.5. Effect of Period Parameters on Bandgap

The period parameter is one of the main factors affecting the bandgap of metamaterials. To further investigate the effect of the period parameter on the bandgap, the dispersion curves of the SPLCRR are calculated for different period lengths and different distribution forms.

**Figure 11:**
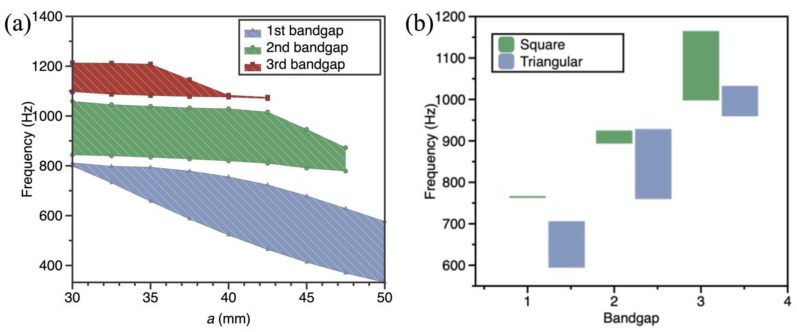
Effect of period parameters on bandgap of the SPLCRR. (**a**) period length (**b**) different distribution forms.

From Figure 11a, it can be obtained that the frequencies of the bandgap are gradually decreasing as the period length increases, which is because the core does not change as the period length increases, resulting in a decrease in the stiffness of the sandwich plate, which causes the bandgap to move to lower frequencies. The width of the first bandgap increases gradually with the period length and then tends to be unchanged. The widths of the second and third bandgaps do not change significantly with increasing period length and then disappear rapidly.

**Figure 12:**
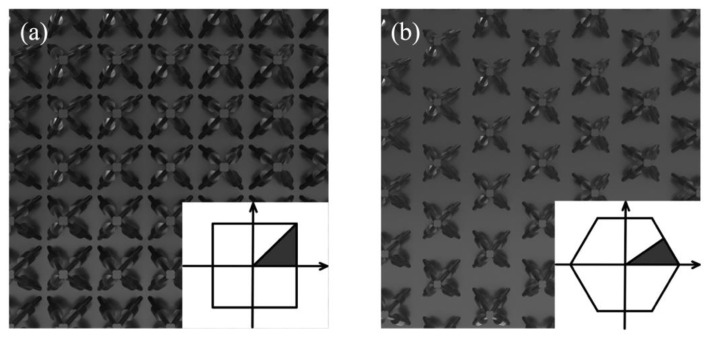
Distribution forms of SPLCRR plate for (**a**) square and (**b**) triangular lattices. First irreducible Brillouin zone in shaded region for (**a**) square and (**b**) triangular lattices.

The dispersion curves of the SPLCRR with different distribution forms are calculated while keeping other parameters constant. The distribution forms are shown in Figure 12a,b. From Figure 11b, it can be obtained that the frequency of the upper and lower edges of the bandgap under the square distribution is higher than that of the hexagonal distribution, where the width of the first and second bandgap of the hexagonal distribution is wider than that of the square distribution, and the opposite is true for the third bandgap. From the effect of the period length on the bandgap in the previous section, it can be seen that the hexagonal distribution increases the distance between the nodes, producing a result that is commensurate with the variation of the period length.

## 4. Conclusions

In this work, inspired by the localized resonant mechanism of classical acoustic metamaterials and considering the integrity of the lattice sandwich plate, we reshaped a sandwich pyramid lattice core with resonant rings (SPLCRR). Based on the experimental verifications and numerical simulations, the effects of geometric parameters, material parameters and period parameters on the bandgaps of the SPLCRR were systematically investigated. Several key observations obtained from the detailed parametric investigation can be summarized as:

(a) Remarkable vibration suppression and bandgaps were verified by comparisons between numerical and experimental results.
(b) Different geometric parameters were discussed. The thickness of face-sheets and the rod radius of the core had significant effects on the frequency range and width of the bandgap, which moved to higher frequency with the increase in the two values. The rod radius was more sensitive to width of the second bandgap compared to other parameters.
(c) Different material parameters were discussed. The elastic modulus of lattice core and face-sheets had significant effects on the frequency range and width of the bandgap, wherein the bandgaps moved to higher frequencies with the increase in the elastic modulus of lattice rod and face-sheets.
(d) Different period parameters were discussed. The frequencies of the bandgap gradually decrease as the period length increases. The frequency of the upper and lower edges of the bandgap under the square distribution was higher than that of the hexagonal distribution.

The research results are expected to be of theoretic significance and offer engineering application prospects.

## Figures and Tables

**Table 1:** Geometric parameters of the SPLCRR.

*a*	*h_c_*	*h_f_*	*h_m_*	*r_c_*	*r_m_*	*θ*
40 mm	38 mm	2 mm	10 mm	2 mm	5 mm	30°

**Table 2:** Material parameters of the SPLCRR [27].

	Density ρ (kg/m^3^)	Modulus *E* (GPa)	Poisson’s Ratio *μ*
Nylon	1200	3.5	0.37
Steel	7800	210	0.3

## Data Availability

All data used to support the findings of this study are available from the corresponding author upon request.

## References

[1] Casalino D., Diozzi F., Sannino R., Paonessa A. (2008). Aircraft noise reduction technologies: A bibliographic review. Aerosp. Sci. Technol..

[2] Ivanov N., Boiko I., Shashurin A. (2017). The Problem of High-Speed Railway Noise Prediction and Reduction. Procedia Eng..

[3] Ma Q., Rejab M.R.M., Siregar J.P., Guan Z. (2021). A review of the recent trends on core structures and impact response of sandwich panels. J. Compos. Mater..

[4] Li Y., Shen C., Xie Y., Li J., Wang W., Cummer S.A., Jing Y. (2017). Tunable Asymmetric Transmission via Lossy Acoustic Metasurfaces. Phys. Rev. Lett..

[5] Sobhani E., Masoodi A.R., Ahmadi-Pari A.R. (2022). Circumferential vibration analysis of nano-porous-sandwich assembled spheri-cal-cylindrical-conical shells under elastic boundary conditions. Eng. Struct..

[6] Li Q., Xie B., Sahmani S., Safaei B. (2020). Surface stress effect on the nonlinear free vibrations of functionally graded composite nanoshells in the presence of modal interaction. J. Braz. Soc. Mech. Sci. Eng..

[7] Fan H., Meng F., Yang W. (2007). Sandwich panels with Kagome lattice cores reinforced by carbon fibers. Compos. Struct..

[8] Fu T., Chen Z., Yu H., Zhu X., Zhao Y. (2019). Sound transmission loss behavior of sandwich panel with different truss cores under external mean airflow. Aerosp. Sci. Technol..

[9] Wang D.-W., Ma L., Wang X.-T., Wen Z.-H., Glorieux C. (2019). Sound transmission loss of laminated composite sandwich structures with pyramidal truss cores. Compos. Struct..

[10] Wen Z.-H., Wang D.-W., Ma L. (2021). Sound transmission of composite sandwich panel with face-centered cubic core. Mech. Adv. Mater. Struct..

[11] Yang G., Hou C., Zhao M., Mao W. (2019). Comparison of convective heat transfer for Kagome and tetrahedral truss-cored lattice sand-wich panels. Sci. Rep..

[12] Fan H.-L., Zeng T., Fang D.-N., Yang W. (2010). Mechanics of advanced fiber reinforced lattice composites. Acta Mech. Sin..

[13] Wu Q., Ma L., Wu L., Xiong J. (2016). A novel strengthening method for carbon fiber composite lattice truss structures. Compos. Struct..

[14] Lu L., Song H., Huang C. (2016). Effects of random damages on dynamic behavior of metallic sandwich panel with truss core. Compos. Part B Eng..

[15] Maury C., Gardonio P., Elliott S.J. (2002). Model for Active Control of Flow-Induced Noise Transmitted Through Double Partitions. AIAA J..

[16] Liu Z., Zhang X., Mao Y., Zhu Y.Y., Yang Z., Chan C.T., Sheng P. (2000). Locally Resonant Sonic Materials. Science.

[17] Cummer S.A., Christensen J., Alù A. (2016). Controlling sound with acoustic metamaterials. Nat. Rev. Mater..

[18] Ding C., Hao L., Zhao X. (2010). Two-dimensional acoustic metamaterial with negative modulus. J. Appl. Phys..

[19] Lee S.H., Park C.M., Seo Y.M., Wang Z.G., Kim C.K. (2009). Acoustic metamaterial with negative modulus. J. Physics Condens. Matter.

[20] Wen Z., Zeng S., Wang D., Jin Y., Djafari-Rouhani B. (2021). Robust edge states of subwavelength chiral phononic plates. Extreme Mech. Lett..

[21] Wen Z., Jin Y., Gao P., Zhuang X., Rabczuk T., Djafari-Rouhani B. (2021). Topological cavities in phononic plates for robust energy harvesting. Mech. Syst. Signal Process..

[22] Wen Z., Wang W., Khelif A., Djafari-Rouhani B., Jin Y. (2022). A perspective on elastic metastructures for energy harvesting. Appl. Phys. Lett..

[23] Park C.M., Lee S.H. (2019). Zero-reflection acoustic metamaterial with a negative refractive index. Sci. Rep..

[24] Choudhury B., Jha R.M. (2013). A Review of Metamaterial Invisibility Cloaks. Comput. Mater. Contin..

[25] Islam S.S., Hasan M.M., Faruque M.R.I. (2018). A new metamaterial-based wideband rectangular invisibility cloak. Appl. Phys. A.

[26] Wang Z., Zhang Q., Zhang K., Hu G. (2016). Tunable Digital Metamaterial for Broadband Vibration Isolation at Low Frequency. Adv. Mater..

[27] Li H., Hu Y., Huang H., Chen J., Zhao M., Li B. (2021). Broadband low-frequency vibration attenuation in 3D printed composite meta-lattice sandwich structures. Compos. Part B Eng..

[28] Li Z.-Y., Ma T.-X., Wang Y.-Z., Li F.-M., Zhang C. (2020). Vibration isolation by novel meta-design of pyramid-core lattice sandwich structures. J. Sound Vib..

[29] An X., Fan H., Zhang C. (2018). Elastic wave and vibration bandgaps in two-dimensional acoustic metamaterials with resonators and disorders. Wave Motion.

[30] Tian H., Wang X., Zhou Y.-H. (2013). Theoretical model and analytical approach for a circular membrane–ring structure of locally resonant acoustic metamaterial. Appl. Phys. A.

[31] Zhang Z., Wei Q., Cheng Y., Zhang T., Wu D., Liu X. (2017). Topological Creation of Acoustic Pseudospin Multipoles in a Flow-Free Sym-metry-Broken Metamaterial Lattice. Phys. Rev. Lett..

[32] Hsu J.-C. (2011). Local resonances-induced low-frequency band gaps in two-dimensional phononic crystal slabs with periodic stepped resonators. J. Phys. Appl. Phys..

[33] Xiao Y., Wen J., Wen X. (2012). Flexural wave band gaps in locally resonant thin plates with periodically attached spring–mass resonators. J. Phys. Appl. Phys..

[34] Miranda E., Nobrega E., Ferreira A., Dos Santos J. (2018). Flexural wave band gaps in a multi-resonator elastic metamaterial plate using Kirchhoff-Love theory. Mech. Syst. Signal Process..

[35] Ruzzene M., Mazzarella L., Tsopelas P., Scarpa F. (2002). Wave Propagation in Sandwich Plates with Periodic Auxetic Core. J. Intell. Mater. Syst. Struct..

[36] Song Y., Feng L., Wen J., Yu D., Wen X. (2015). Reduction of the sound transmission of a periodic sandwich plate using the stop band concept. Compos. Struct..

[37] An X., Lai C., Fan H., Zhang C. (2020). 3D acoustic metamaterial-based mechanical metalattice structures for low-frequency and broadband vibration attenuation. Int. J. Solids Struct..

